# The role of D-serine as co-agonist of NMDA receptors in the nucleus accumbens: relevance to cocaine addiction

**DOI:** 10.3389/fnsyn.2014.00016

**Published:** 2014-07-16

**Authors:** Marcello D’Ascenzo, Maria Vittoria Podda, Claudio Grassi

**Affiliations:** Institute of Human Physiology, Medical School, Universitá Cattolica “S. Cuore”Rome, Italy

**Keywords:** addiction, cocaine, D-serine, NMDA receptors, nucleus accumbens, synaptic plasticity

## Abstract

Cocaine addiction is characterized by compulsive drug use despite adverse consequences and high rate of relapse during periods of abstinence. Increasing consensus suggests that addiction to drugs of abuse usurps learning and memory mechanisms normally related to natural rewards, ultimately producing long-lasting neuroadaptations in the mesocorticolimbic system. This system, formed in part by the ventral tegmental area and nucleus accumbens (NAc), has a central role in the development and expression of addictive behaviors. In addition to a broad spectrum of changes that affect morphology and function of NAc excitatory circuits in cocaine–treated animals, impaired N-methyl-D-aspartate receptor (NMDAR)-dependent synaptic plasticity is a typical feature. D-serine, a D-amino acid that has been found at high levels in mammalian brain, binds with high affinity the co-agonist site of NMDAR and mediates, along with glutamate, several important processes including synaptic plasticity. Here we review recent literature focusing on cocaine-induced impairment in synaptic plasticity mechanisms in the NAc and on the fundamental role of D-serine as co-agonist of NMDAR in functional and dysfunctional synaptic plasticity within this nucleus. The emerging picture is that reduced D-serine levels play a crucial role in synaptic plasticity relevant to cocaine addiction. This finding opens new perspectives for therapeutic approaches to treat this addictive state.

## Introduction

A large body of evidence has demonstrated the fundamental role of D-serine as co-agonist at the N-methyl-D-aspartate receptor (NMDAR), a major glutamate receptor subtype involved in synaptic plasticity (Mothet et al., 2000; Panatier et al., 2006; Fossat et al., 2012; Rosenberg et al., 2013). In particular, D-serine, by binding the so called “glycine site” on the NR1 subunit, is crucial for the activation of these receptors and for NMDAR-dependent synaptic plasticity mechanisms. D-serine degradation by the FAD-dependent enzyme D-amino acid oxidase (DAAO) suppresses NMDAR-dependent component of excitatory synaptic currents and prevents NMDAR-dependent long term potentiation (LTP) and depression (LTD) in different brain areas, including the hippocampus, the supraoptic nucleus and prefrontal cortex (Yang et al., 2003; Panatier et al., 2006; Fossat et al., 2012).

Abnormal levels of D-serine have been reported in aging (Potier et al., 2010; Turpin et al., 2011; Haxaire et al., 2012; Billard, 2013), Alzheimer’s disease (Wu et al., 2004) and amyotrophic lateral sclerosis (ALS; Sasabe et al., 2012; Paul and de Belleroche, 2014). Moreover, D-serine has emerged as an influential player in the context of psychiatric diseases such as schizophrenia and depression. Indeed, based on the rationale that a common feature of these pathologies might be a dysregulation of glutamatergic system, especially NMDAR-dependent synaptic transmission, an increasing number of studies have investigated D-serine signaling to explore possible causes and potential therapeutic interventions for these diseases (Carlsson and Carlsson, 1990; Adage et al., 2008; Lisman et al., 2008; Gunduz-Bruce, 2009; Hashimoto et al., 2009; Labrie et al., 2012; Balu et al., 2013; Lane et al., 2013; Sacchi et al., 2013).

More recently a link between D-serine signaling and cocaine addiction, another neuropsychiatric disorder, has been proposed. Cocaine addiction is a pathological learned behavior characterized by compulsive drug seeking and high vulnerability to relapse even after prolonged abstinence (Mendelson and Mello, 1996). A large body of evidence has linked the development and expression of this addictive behavior to neuroadaptations in the mesocorticolimbic system. The interconnected brain regions that make up this system include the prefrontal cortex, the ventral tegmental area, and the ventral striatum (also known as nucleus accumbens, NAc), which has been critically implicated in the expression of a variety of addiction-related behavioral alterations (Koob and Volkow, 2010). In particular, cocaine-induced neuroadaptations in the NAc have been associated with changes in glutamatergic synaptic transmission and plasticity (Kauer and Malenka, 2007; Lüscher and Malenka, 2011). In this context, accumulating evidence indicates an impairment of NMDAR-dependent LTP and LTD at glutamatergic synapses in the NAc of animal models of cocaine addiction (Martin et al., 2006; Moussawi et al., 2009; Kasanetz et al., 2010).

A link between D-serine and cocaine addiction was first indicated by findings that D-serine reduced the expression of cocaine-induced conditioned place preference (a standard behavioral model used to study the rewarding and aversive effects of drugs) (Yang et al., 2013), and that reduced cocaine-primed reinstatement following extended access to cocaine self-administration (Kelamangalath and Wagner, 2010). Within this context we have recently addressed the issue of whether D-serine signaling could be involved in cocaine-induced neuroadaptations in the NAc of a rat model of non-contingent (passive) cocaine exposure (Curcio et al., 2013).

### D-serine-related pathway in the NAc

Since the initial detection of D-serine in the central nervous system (Hashimoto and Oka, 1997) subsequent immunostaining studies have more precisely localized D-serine, DAAO and serine racemase (SR), the major biosynthetic enzyme for D-serine, in mammalian brain (Williams et al., 2006; Verrall et al., 2007; Paul and de Belleroche, 2014). SR was initially identified in astrocytes and microglia (Wolosker et al., 1999; Stevens et al., 2003; Wu et al., 2004; Panatier et al., 2006). More recently, however, SR was also identified in neurons (Kartvelishvily et al., 2006; Dun et al., 2008; Wolosker, 2011; Miya et al., 2008; Rosenberg et al., 2013; Balu et al., 2014) challenging the classical view that D-serine is a gliotransmitter.

In the NAc the presence of D-serine, DAAO and SR was found in both neurons and astrocytes (Curcio et al., 2013). This cellular localization in the NAc is consistent with the model of D-serine dynamics proposed by Wolosker (2011) that is based upon the notion that D-serine is primarily made in neurons and explains why drugs blocking astrocyte metabolism affect D-serine extracellular level (Zhang et al., 2008; Henneberger et al., 2010). This so-called “serine shuttle model” proposes that astrocytes synthesize and export L-serine to neurons to fuel the synthesis of D-serine by the neuronal SR. Once synthesized and released by neurons, D-serine can be taken up by astrocytes for storage and activity-dependent release (Martineau et al., 2013). Our results also showed that the intensity of immunostaining for D-serine and its related enzymes among labeled neuronal cells (>80% of NAc core neurons) was different, likely reflecting different levels of expression. Considering that there are two main types of MSNs: those expressing D1-like receptors and those that express D2-like dopamine receptors (Sesack et al., 1994; Hara and Pickel, 2005; D’Ascenzo et al., 2009; Podda et al., 2010), and that these cell subtypes show different responses to cocaine exposure (Lobo et al., 2010; Maze et al., 2014), it would be of interest to establish whether these two neuronal subtypes differentially express D-serine machinery.

As in other brain areas, the presence of D-serine in the NAc is related to its ability to act as NMDAR co-agonist at synaptic level. Indeed, electrophysiological data provided by our group showed a marked increase in AMPA/NMDA ratio at excitatory synapses in brain slices where D-serine levels had been lowered by incubation with *Rg*DAAO. In contrast enzymatic degradation of glycine with glycine oxidase did not affect AMPA/NMDA ratio (Curcio et al., 2013). The same study also revealed the essential role of D-serine in synaptic plasticity mechanisms within the NAc by showing that enzymatic D-serine degradation prevented the induction of NMDAR-dependent LTP and LTD.

### Reduced D-serine levels in a rat model of cocaine-addiction

Although our knowledge on the molecular mechanisms underlying the behavioral changes induced by cocaine abuse is far from be complete, research on rodent models has successfully identified alterations in NMDAR-dependent synaptic plasticity as an important component of these behavioral changes.

Several studies have indeed demonstrated impairment of LTP and LTD in different models of cocaine addiction. *In* *vivo* experimental evidence showed that after long-term withdrawal from cocaine self-administration LTP cannot be induced in the rat NAc core (Moussawi et al., 2009). The impairment of LTP reported in this study has been attributed to a pre-existing LTP-like state, developed during cocaine treatment and/or withdrawal, in which the synaptic strength is maximized. Moussawi et al. (2009) also observed loss of LTD inducibility in the rat NAc after long-term withdrawal from self-administered cocaine, an effect that could not be explained by pre-existing synaptic potentiation. Impairment of LTD has also been reported by other authors in rats with a history of cocaine in both *in* *vivo* and *ex vivo* (i.e., brain slices) preparations (Martin et al., 2006; Kasanetz et al., 2010). It is well known that in many regions of the brain the induction of both LTP and LTD is dependent on the activation of NMDARs which leads to postsynaptic Ca^2+^ influx. LTP is mediated by large, short-lived increases in the intracellular Ca^2+^ concentration that is believed to occur following activation of synaptic NR2A-containing NMDARs, whereas moderate Ca^2+^ increases through extrasynaptic NR2B-containing NMDARs have been involved in the induction of LTD (Massey et al., 2004; Yashiro and Philpot, 2008; Hunt and Castillo, 2012). Such different regulation of intracellular Ca^2+^ levels are supposed to trigger different subset of Ca^2+^-dependent intracellular signaling molecules required for both forms of synaptic plasticity.

NMDAR hypofunction with reduced Ca^2+^ influx at the postsynaptic level is an attractive hypothesis to explain the impairment of both LTP and LTD in cocaine-treated animals.

**Figure 1:**
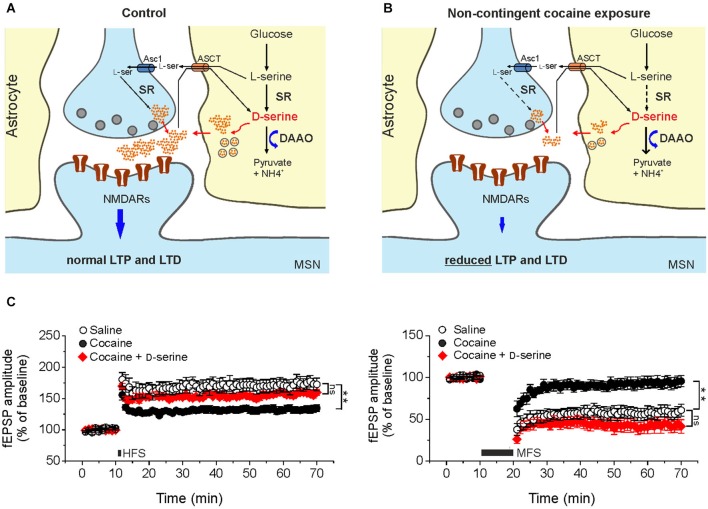
**Cocaine-induced deficits in NMDAR-dependent synaptic plasticity in the NAc result from reduced D-serine levels**. Compared to control animals **(A)** in cocaine-treated rats **(B)** reduced levels of D-serine at glutamatergic synapses impinging on medium spiny neurons leads to NMDAR hypofunction that, in turn, causes impairments of LTP and LTD elicited by standard stimulation protocols. Dashed arrows in **B** indicate reduced expression of the enzyme whereas thick arrows indicate increased levels of the enzyme. Asc1, alanine-serine-cysteine-1 transporter-1. ASCT, alanine/serine/cysteine/threonine transporter. **(C)** Time course of LTP and LTD in NAc slices from saline- (control) and cocaine-treated rats. High frequency stimulation (HFS) and medium frequency stimulation (MFS) inducing LTP (left) and LTD (right), respectively, are impaired in slices from cocaine-treated rats, but not when the medium is supplemented with D-serine (red, ** *p* < 0.001). Data are adapted from Curcio et al. (2013).

In keeping with this, recent findings from our laboratory have raised the intriguing possibility that cocaine treatment is associated with changes in D-serine signaling, that contribute to synaptic plasticity dysfunction (Figure 1). In slices from cocaine-treated rats exposure to saturating levels of exogenous D-serine fully restored LTP and LTD inducibility (Figure 1C). Moreover, NMDAR neurotransmission driven by endogenous D-serine was impaired in the NAc slices from cocaine-treated rats (Curcio et al., 2013).

Further support to the involvement of NMDAR hypofunction in mediating changes in synaptic plasticity observed in our cocaine treatment paradigm comes from data demonstrating an increase in AMPA/NMDA ratio in cocaine-treated rats that was not associated with changes in AMPA receptor function and expression. Additionally, biochemical assays revealed that D-serine levels in the NAc extract obtained from cocaine-treated rats were significantly lower than those found in extracts from saline-treated rats.

The molecular determinants of D-serine synaptic turnover affected by cocaine treatment has yet to be definitively identified. However, considering that DAAO expression was increased in cocaine treated rats at both the transcriptional and post-transcriptional levels, whereas SR expression was reduced only at post-transcriptional level, it may be speculated that the main contribution to cocaine-related decrease in D-serine content arises from both increased degradation and decreased synthesis (Figure 1B; Curcio et al., 2013).

Although molecular and functional data from our and other studies established a clear link between impaired D-serine signaling and altered LTP and LTD in the NAc, the understanding of cocaine–induced neuroplasticity changes is a very complex and debated issue since different neuroadaptive changes at molecular and cellular levels can occur at the same time in the NAc (Nestler, 2005; Kreek et al., 2012). Furthermore, adding complexity to this scenario, conflicting results have been reported depending on the drug-exposure protocols (e.g., self-administration or passive), animal models used and duration of withdrawal (e.g., short or long-term) (Thomas et al., 2001; Kourrich et al., 2007; Bowers et al., 2010; Wolf and Tseng, 2012; Ortinski et al., 2013).

### Reduced D-serine signaling in the NAc as molecular correlate for cocaine-induced locomotor sensitization

Locomotor sensitization (usually measured as increased locomotor responses to repeated administration of cocaine) has been related to impairment of synaptic plasticity in the NAc (Thomas et al., 2001; Kalivas et al., 2009). Moreover, this behavioral paradigm has provided a major impetus to explore the neuroplasticity that may occur during the transition from drug use to addiction (Koob and Volkow, 2010) and several studies have demonstrated the essential role played by the NAc in both the induction and expression phases of this cocaine-induced behavioral alteration (Kalivas et al., 2009).

**Figure 2:**
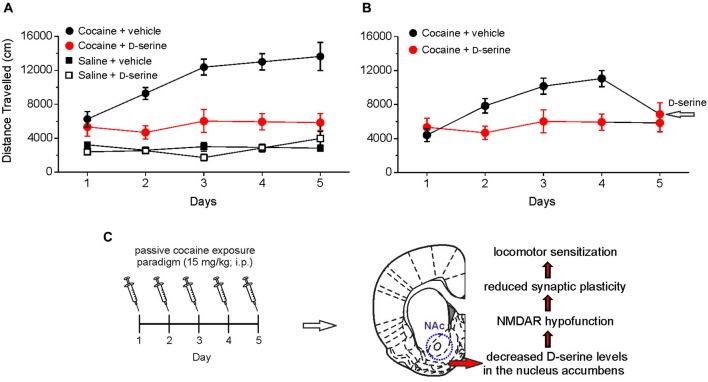
**Intra-accumbens microinjection of D-serine blocks locomotor sensitization, an hallmark behavioral feature associated with chronic exposure to cocaine. (A)** Analysis of locomotor activity measured by using an open field apparatus showing the distance traveled by different groups of rats treated with saline or cocaine (15 mg/kg, intraperitoneal, i.p.) and receiving intra-accumbens D-serine (0.4 μg/0.3 μl) or vehicle. When rats are intra-NAc microinjected with D-serine prior to cocaine treatment (red), the development of cocaine sensitization is blocked (i.e., the distance traveled on days 2–5 is not significantly different from day 1). **(B)** A single intra-NAc injection of D-serine at day 5 reverts the effect of cocaine on locomotor activity (days 1–4). **(C)** Summary of cocaine-induced D-serine signaling dysregulation in the NAc and consequent functional alterations. Data are taken from Curcio et al. (2013).

By injecting D-serine directly into the NAc, we demonstrated that this amino acid blocks the development of locomotor sensitization to cocaine (Figure 2). These findings indicate that cocaine-induced impairment of D-serine signaling may be a molecular correlate for cocaine-induced behavioral sensitization and strongly support the notion that NMDAR-associated glycine recognition site plays an important role in the cocaine induced behavioral changes. In keeping with this, it has been reported that partial agonists at NMDAR glycine site have beneficial effects in rescuing cocaine–induced behavioral alterations (Khan and Shoaib, 1996; Huang et al., 2008; Thanos et al., 2009; Nic Dhonnchadha et al., 2010; Torregrossa et al., 2010). The finding that D-serine reverts both the cocaine-induced synaptic plasticity impairment and locomotor sensitization points to this amino acid as an attractive tool for counteracting the behavioral changes induced by cocaine in humans. However, in this respect it should be pointed out that, although still under debate (D’Souza et al., 2013), the use of long-term treatment with large doses of D-serine has been reported to cause side-effects, especially the oxidation-dependent necrosis of renal proximal tubules (Williams and Lock, 2004; Krug et al., 2007). A possible strategy to overcome potential side effects would be to employ low doses of D-serine together with oral administration of DAAO inhibitors. This therapy has been successfully used to reduce cognitive symptoms in animal models of schizophrenia (Adage et al., 2008; Ferraris et al., 2008; Hashimoto et al., 2009). Furthermore, a recent study showed that sodium benzoate, a DAAO inhibitor, *per se* significantly improved a variety of symptom domains and neurocognition in patients with chronic schizophrenia (Lane et al., 2013). Taken together these findings hold promise for treating cocaine addiction by targeting D-serine signaling.

## Conclusions

Cocaine is the second most commonly used illegal drug worldwide after cannabis. Prevalence of cocaine use (last year, lifetime) is particularly high among aged between 15 and 34 years. Cocaine addiction is a worldwide public health problem with consequences beyond its somatic and psychiatric effects including socio-economic and judicial complications. The number of long-term cocaine-dependent patients entering drug treatment has been increasing in Europe for several years. However, there is no specific pharmacotherapy with established efficacy for the treatment of cocaine addiction and basic research on this field represents one of the most important tool to face and counteract this important public health problem.

During the last 5 years data have accumulated assigning a significant role to D-serine in mediating synaptic plasticity and behavioral changes associated with cocaine addiction. These findings encourage the development of new pharmacological interventions targeting D-serine signaling to treat addiction. However, many questions still remain to be answered, especially those regarding the synaptic turnover of this amino acid in the brain. For example, what is the selective contribution of astrocytes and neurons to D-serine release? Is D-serine released through vesicular and/or nonvescicular mechanisms? How SR and DAAO expression and activity are regulated? The understanding of these mechanisms is essential before considering the development of D-serine-derived drugs for the treatment of cocaine addiction and other psychiatric disorders or cognitive deficits associated to impaired NMDAR-dependent synaptic plasticity.

## Conflict of interest statement

The authors declare that the research was conducted in the absence of any commercial or financial relationships that could be construed as a potential conflict of interest.
